# Case of Hysteric Phrenitis, Supervening on Pulmonary Inflammation

**Published:** 1805-11-01

**Authors:** J. A. Hendy

**Affiliations:** Fellow of the Edinburgh Medical Society


CASE *of* HYSTERIC PHRENITIS, *supervening on* PULMONARY INFLAMMATION; *by* J. A. HENDY, M. D. Fellow of the Edinburgh Medical Society.

ON the 1st of February I was sent for to visit Benjamin Dawson, aged 32, of the parish of Boreham, three miles distant from Chelmsford. He had been seized six days before with the common symptoms of pneumonic inflammation, for the removal of which he had been twice bled, blistered, and in other respects judiciously treated by Mr. Cremer, an accurate and attentive practitioner of this town.

On entering the room, not being apprized of such morbid conversion, I was much surprised to find my patient in a state of wild delirium, using violent exertions to get out of bed, expressing great disgust, and exhibiting, in consequence of the restraint imposed upon him, considerable resistance.

The countenance was distorted and anxious, and vision evidently imperfect, from the irregular direction of the hands, in his attempts to lay hold of the attendants confining him to bed. There *appeared* no one symptom of pulmonic affection. The pulse was, as nearly as I could ascertain, 140 or more, small and irregular; skin hot; body costive; no expectoration, but a constant spitting of frothy saliva on himself, and the objects around him. My visit was made at two o'clock. I understood that the alteration of symptoms had come on suddenly about eleven o'clock, and that since that time he had remained precisely in the state he then was. I ordered a glyster to be immediately administered; the head to be shaved and blistered; the feet to be put in warm water, and a saline anodyne antimonial draught to be given at bed-time. I dined in the neighbourhood, and on my return home, between six and seven in the evening, I again saw the patient.

The countenance seemed much depressed; he was in a state of complete exhaustion, not sensible; but on being roused, evinced a partial recollection of particular persons.

Fæces and urine had been passed in bed; cough was now present, but without any expectoration; respiration extremely quick (48 in the minute), and very stridulous; the pulse weak, and as frequent as in the morning. Much tremor of the tongue, which was dry and furred. I gave him the night draught, and promised to see him in the morning.

Feb. 2d. He had slept much during the night, and had been in a profuse general perspiration, which was still present; there was great pain of chest, frequent cough, with very slight expectoration; pulse and respiration nearly as yesterday; body costive; he evinced considerable anxiety of mind, marked by sobbing, and a strong disposition to weep. Blisters on the head and ankles had risen well, but had produced strangury to a painful degree. He was ordered to dilute freely; an injection to be administered, and the following medicines, with the use of Mudge's Inhaler, were prescribed.

℞. Kali ppt. ʒij; succi limonis recentis ℥iij vel q. s. ad saturandum alkali; mellis acetati ℥j; vini ipecacuanhæ ʒiij; tinct. digitalis saturatæ, gtt. 120; aquæ fontis ℥xij; M. sumat coch. iij ampla, 3tiâ quâque horâ. Rep. haustus nocturnus, omissâ tincturâ opii; sit emplast. cantharidis sterno.

3d. He had some sleep during the last night; the cough had been very frequent; expectoration pretty copious and thick; orthopnœa still considerable, and he can lie only on his back; skin hot, but covered with a profuse general perspiration, which came on in the evening, and continued during the whole of the night. He has shewn great uneasiness of mind, and has had repeated bursts of tears, after which he has appeared more composed; he drinks plentifully, and finds the use of the inhaler soothing and comfortable; there is occasional sopor, and imperfect recollection; p. 126, weak, but regular; the urine is copiously secreted. Body open.

Persistat in usu misturæ et haustus.

4th. The patient was visited on this day by Mr. Cremer, who informed me that the cough, expectoration, and stertorous breathing were nearly as yesterday; that the skin was cooler and more relaxed; the pulse about 86, and natural, and that he lay in a state of drowsiness bordering on insensibility, with dilated pupil, uninfluenced by the immediate application of a lighted candle.

Mr. Cremer proposed ordering a mixture with the compound spirit of vitriolic æther, to which I readily agreed. Before it had been sent, in the evening of this day, I was called on by my friend Mr. Ray, the clergyman of Boreham, who described the man as dying; he said, that soon after Mr. Cremer had left him, the lower jaw had become clenched, and the teeth so strongly closed as to deny ingresss to medicine or any fluid; that the expectoration had ceased, the countenance sunk, eyes fixed, and insenible sible to every external impression. I felt disappointed at this report, but I was willing to hope that the case, not viewed medically, might be rendered more formidable than it really was, through the benevolent medium of pastoral anxiety. With the use of the mixture of Hoffman's anodyne, I ordered a liniment, with compound soap liniment, tincture of cantharides and laudanum, to be well rubbed into the internal fauces, and sinapisms to be applied to the feet; in which, however, I had been anticipated by Mr. Cremer.

5th. A sudden alteration of the jaw took place last night, to which copious expectoration supervened. He had some sleep during the night; tongue foul, but moist; countenance more cheerful; breathing still difficult and stridulous; pain of chest less severe; skin soft, and covered with general warm perspiration; cough less frequent; he has been able to lie on his side, though not with perfect ease; body costive; p. 80, soft and æquable. Vespere injic. enema laxans.

Omitt. mist. c. tinct. digitalis saturatâ. ℞. Misturæ mucil. g. arabici; aquæ rosar. damas. aa. ℥iijß; vini ipecacuanhæ ʒiij; aceti scillit ʒij; mellis acetati ℥ß; M. sumat. coch. ij. ampla, ex cyatho aquæ hord. 3tiâ quâque horâ.

℞. Vini antimonii gtt .xx; aquæ ammoniæ acetatæ ℥ß; mellis acetati ℥iß; mist. camphor. aquæ fontis aa ℥ß: m. sit pro haustu, noctu dormituro sumend.

7th. There is a gradual and marked remission of pain, dyspnœa, and cough; complains of weakness, and expresses a desire for food. A light nourishing diet was now allowed, and the medicines continued.

10th. In every respect he is better. There is still some cough, and the more urgent paroxyms excite slight pain of side; respiration nearly natural, and unmarked by the stertorous noise, which has accompanied it throughout; functions natural.

Omitt. haustus. nocturnus. Rep. mistura.

13th. Convalescent.

The mixture ordered on the 5th was continued for some days beyond his convalescence, He was afterwards ordered a mucilaginous mixture acidulated with vitriolic acid; and recovering his strength but very slowly, he afterwards used the common myrrh and steel mixture of the late Dr. Griffiths. To the date of this communication, Dawson has enjoyed the most perfect health; the epileptic fits, which were before perpetually harrassing him, have entirely tirely disappeared; and since his illness, he has had no one threatening of their invasion.

### REMARKS.

In every point of view, the fortunate termination of poor Dawson's illness, afforded infinite gratification to my mind. In the humble sphere of life in which he is placed, he may be considered as a most useful member of that class to which he belongs. A good father, a good husband, and an industrious man, involve points which attach value to the life of such an individual. In a practical point of view too, the effect of medicines in combating the violence of symptoms was encouraging and satisfactory; considered abstractedly, as a case of severe pneumonia, where the pathology is so clearly and distinctly marked, and the mode of treatment so unequivocally pointed out, it might be deemed superfluous to intrude it upon medical attention, beyond the sphere which took cognizance of it; but the variations which it assumed during its progress, fixed my attention to its more particular re-consideration, and have induced me to give the detail, which I hope may not be altogether uninteresting to your readers.*

On first seeing the patient, I unequivocally pronounced him to be in a state of the most extreme danger. I viewed the affection of the brain as indisputably depending on metastasis of the inflammation, from the lungs to that organ; and under this impression I looked forward to the almost uniform consequence, dissolution.

Encouragement was not to be drawn from the naturally weak and delicate constitution of the man, added to the circumstance of his being long afflicted with epilepsy.

On my second visit, in the evening of the first, my attention was naturally drawn to the sudden recession of the phrenitic, and the revisitation of the pulmonic symptoms; the last, under appearances the most formidable. Viewed physiologically, their co-existence would have presented nothing incompatible with the known laws of the animal œconomy. The presence of true and genuine pneumonia, from its commencement to its temporary suspension, and again

* Symptomatic phrenitis coming on, upon the ceasing or spreading of pulmonary inflammation, is likewise an event neither new nor extraordinary; the cough and dyspnœa causing the determination to the head, would of themselves explain such an occurrence; but the whole ratio symptomatum in pneumonia, is clear and satisfactory.

again onward to the close, was sufficiently ascertained; every symptom was regular, and in strict conformity with nosological definition. Not so with the supervening disease; I doubted whether *it* could be considered as an instance of *real* phrenitis, properly so called, occurring either by conversion or extension of the pre-existing inflammation. Many circumstances strengthened me in such scepticism; the unevenness of disposition, the convulsive sobs and strong paroxysms of weeping continuing for many hours after all the symptoms of irritated brain had subsided, induced me rather to suppose that this *exact representation* of phrenitic delirium, might be referred to the *imitative* dexterity of an *hysteric paroxysm*. Such a conjecture is at least admissible, whether we view the symptoms uniformly attached to phrenitis, its progress, termination, &c. or whether we take into the account the "versatile" character of metamorphic hysteria. Such considerations applied to, and compared with the present history, would certainly induce me to add this to the many cases on record, where hysteria has been known to assume (without danger to life) the leading features of distinct diseases, in *themselves* universally fatal, when uninfluenced by nervous counterfeit. I would here refer the reader to Dr. Ferriar's very valuable paper, "on the Conversion of Diseases," in the second vol. of his "Medical Histories and Reflections." Its perusal formerly made a strong impression on my mind, and a recurrence to the very valuable, scientific, and practical remarks which it contains, may probably have occasioned me to take the present view of the case now under consideration. I shall make no apology for quoting at length Dr. Ferriar's remarks, introductory to his detail of the particular instances of masked hysteria, primary, and supervening to other diseases.

Speaking of hysteric conversion, he thus expresses himself: "Whoever would present us with a good book on the fallacy of symptoms, which is much wanted, must be completely master of this unaccountable disease."

"We are ignorant by what laws the body possesses a power of representing the most hazardous disorders, without incurring danger; of counterfeiting the greatest derangement in the circulating system, without materially altering its movements; of producing madness, conscious of its extravagancies, and of increasing the acuteness of sensation by oppressing the common sensorium. In hysterical affections, all these appearances are excited, which are incompatible with the reasonings of every system-maker, maker, who has yet endeavoured to explain the inexplicable. Nature, as if in ridicule of the attempts to unmask her, has in this class of diseases, reconciled contradictions, and realised improbabilities, with a mysterious versatility, which inspires the true philosopher with diffidence, and reduces the systematic to despair."

The symptoms of torpor, occurring on the 4th, may have been much influenced by the action of the Fox Glove, (he had taken four bottles of the mixture) but even these were probably modified and rendered more formidable in appearance by hysteric determination.

It does not appear that the *progress* of the pulmonary affection was materially influenced, beyond its temporary suspension, by the accession of the new train of symptoms. Can we suppose the hysteric paroxysm to have been critical, and conducive to the happy termination of the primary disease, the menacing symptoms of which began gradually to subside afterwards, with evident marks of remission, and copious discharges by the skin, lungs, &c. leading on to complete resolution of the disease.

The morbid conversion can scarcely be viewed as an *accidental* occurrence, invading while under other diseased action, a subject, where the pre-existence of strongly marked hysteric predisposition seems to have been amply confirmed.

The entire subject admits of further discussion, but I feel that I have already sufficiently encroached on the pages of your Journal.

Without entering on the modus operandi of the medicines employed, they must be allowed (detached from all theoretical reasoning), to have exerted a salutary and decided influence; and abstaining from all further comment, I close the history of a case already perhaps too prolix.

*Chelmsford, Sept. 6, 1806.*

