# Errata

**Published:** 1805-12

**Authors:** 


### ERRATA.

Page 393, line 8, *for* internal *read* external.

line 25, *for* ℥ifs, *read* ʒifs.

---

END OF VOL. XIV.

